# Metabolic syndrome: epidemiology, mechanisms, and current therapeutic approaches

**DOI:** 10.3389/fnut.2025.1661603

**Published:** 2025-09-03

**Authors:** Benson M. Hamooya, Lukundo Siame, Lweendo Muchaili, Sepiso K. Masenga, Annet Kirabo

**Affiliations:** ^1^Department of Public Health, School of Medicine and Health Sciences, Mulungushi University, Livingstone, Zambia; ^2^Department of Cardiovascular Science and Metabolic Diseases, Livingstone Center for Prevention and Translational Science, Livingstone, Zambia; ^3^Vanderbilt Institute for Global Health, Vanderbilt University Medical Center, Nashville, TN, United States; ^4^Department of Medicine, Vanderbilt University Medical Center, Nashville, TN, United States; ^5^Vanderbilt Center for Immunobiology, Vanderbilt University Medical Center, Nashville, TN, United States; ^6^Vanderbilt Institute for Infection, Immunology and Inflammation, Vanderbilt University Medical Center, Nashville, TN, United States

**Keywords:** metabolic syndrome, cardiovascular disease, dyslipidemia, insulin resistance, inflammation, oxidative stress, obesity, hypertension

## Abstract

Metabolic syndrome (MetS) is a complex condition marked by central obesity, dyslipidemia, hypertension, insulin resistance, oxidative stress, and chronic inflammation. These risk factors significantly raise the risk of cardiovascular disease (CVD) through various mechanisms, leading to a public health challenge. MetS contributes to CVD through cardiometabolic derangements such as endothelial dysfunction, atherosclerosis, oxidative stress, and inflammation. Dyslipidemia, especially elevated triglycerides and reduced high-density lipo-protein (HDL) cholesterol is central to atherosclerosis. Additionally, hypertension and insulin resistance damage blood vessels, a process exacerbated by chronic inflammation and oxidative stress. Thus, managing MetS and its components through lifestyle changes like weight control, dietary improvements, exercise, and smoking cessation is essential for reducing CVD risk. Medications targeting specific risk factors, such as blood sugar, cholesterol, and blood pressure, may also be required. Raising awareness and early screening are crucial to countering MetS’s impact on public health. This review provides a comprehensive overview of the mechanisms by which MetS contributes to CVD and the intricate interplay of factors and molecular pathways linking MetS to CVD.

## 1 Introduction

Metabolic syndrome (MetS) is a collection of metabolic abnormalities that include central obesity, dyslipidemia, hypertension, and insulin resistance (IR), first observed and named syndrome X by the American endocrinologist Gerald Reaven in 1988. He named it syndrome X to describe the interconnectedness of the components of the syndrome to cardiovascular disease (CVD) (1). The underlying pathophysiology of MetS is primarily intertwined with both genetic and environmental factors that collectively contribute to IR and chronic low-grade inflammation (2).

Metabolic syndrome has been extensively linked to an elevated risk of developing serious non-communicable diseases, notably cardiovascular disease (CVD), stroke, and type 2 diabetes (T2DM) (3). These health conditions are responsible for a significant number of deaths in Western countries and are increasingly becoming prevalent in the developing world as well. This can be attributed, in part, to the adoption of Western lifestyle habits, characterized by a calorie-rich diet, sedentary behavior, and reduced manual labor (3).

However, the precise pathophysiological mechanisms underlying MetS remain incompletely understood. While some researchers emphasize IR as the primary causal factor, others posit inflammation, oxidative stress, mitochondrial dysfunction or nutritional imbalances as contributing factors (2, 4, 5). Additionally, there is debate surrounding the role of obesity as the central determinant of MetS, with some scholars proposing ectopic fat accumulation, adipose tissue dysfunction, or lipotoxicity, all of which are strongly influenced by dietary patterns as potential driving forces (6).

The central objective of this comprehensive review is to provide a thorough overview of MetS, with particular emphasis on its nutritional determinants and modifiable dietary risk factors. In doing so, the review explores the epidemiology, underlying mechanisms, and current dietary and lifestyle-based management strategies by synthesizing existing knowledge and highlighting key discoveries, this review aims to illuminate the complexities of MetS and guide future interventions and research directions.

## 2 Epidemiology of metabolic syndrome

The prevalence of metabolic syndrome varies widely across different populations and age groups. According to the US National Health and Nutrition Examination Survey (NHANES) 2011-18, approximately 39.8% of the US adult population meets the criteria for metabolic syndrome, with the prevalence increasing with age. Among adults aged 20–39, about 22.2% are affected, and increases to 56.4% in those aged 60 and above (7). MetS is also more common in certain ethnic groups; for instance, Hispanic Americans have the highest prevalence compared to their Caucasian and African-American counterparts (8).

The prevalence of MetS in China is estimated to be 24.2%, with older participants and women having increased odds of having MetS (9). Chinese of Korean ethnicity have increased odds of having MetS compared to Chinese of Tibetan and other ethnicities (10).

In Africa, MetS prevalence is approximated to be at 32.4% in the adult population, with older participants and women having increased odds of having the syndrome (11). There has been a notable increase in the prevalence of MetS in Africa over the past few years and it has been attributed to several factors including HIV antiretroviral therapy in people living with HIV (PLWH), and changing lifestyles and diets to adopt Western lifestyles and diets in the general population (12, 13).

Globally, the overall prevalence of metabolic syndrome is estimated to be around 25%, with variations attributed to genetic, environmental, and lifestyle factors (14, 15). While traditionally developed countries have had a higher prevalence of MetS, there is a general upward trend in developing countries (16, 17). There has also been a notable global increase in MetS-associated deaths (18, 19).

The impact of MetS extends beyond individual health; it imposes a substantial economic burden on healthcare systems. The management of MetS and its complications, such as CVD and diabetes, requires considerable healthcare resources. This includes costs related to medical treatments, hospitalizations, and loss of productivity due to illness (20).

## 3 Risk factors of metabolic syndrome

Previous literature has shown a strong association between MetS and the changes in fat or adipocyte function, HIV, older age, high viral load, and CD4 counts less than 350 (21–27).

**FIGURE 1:**
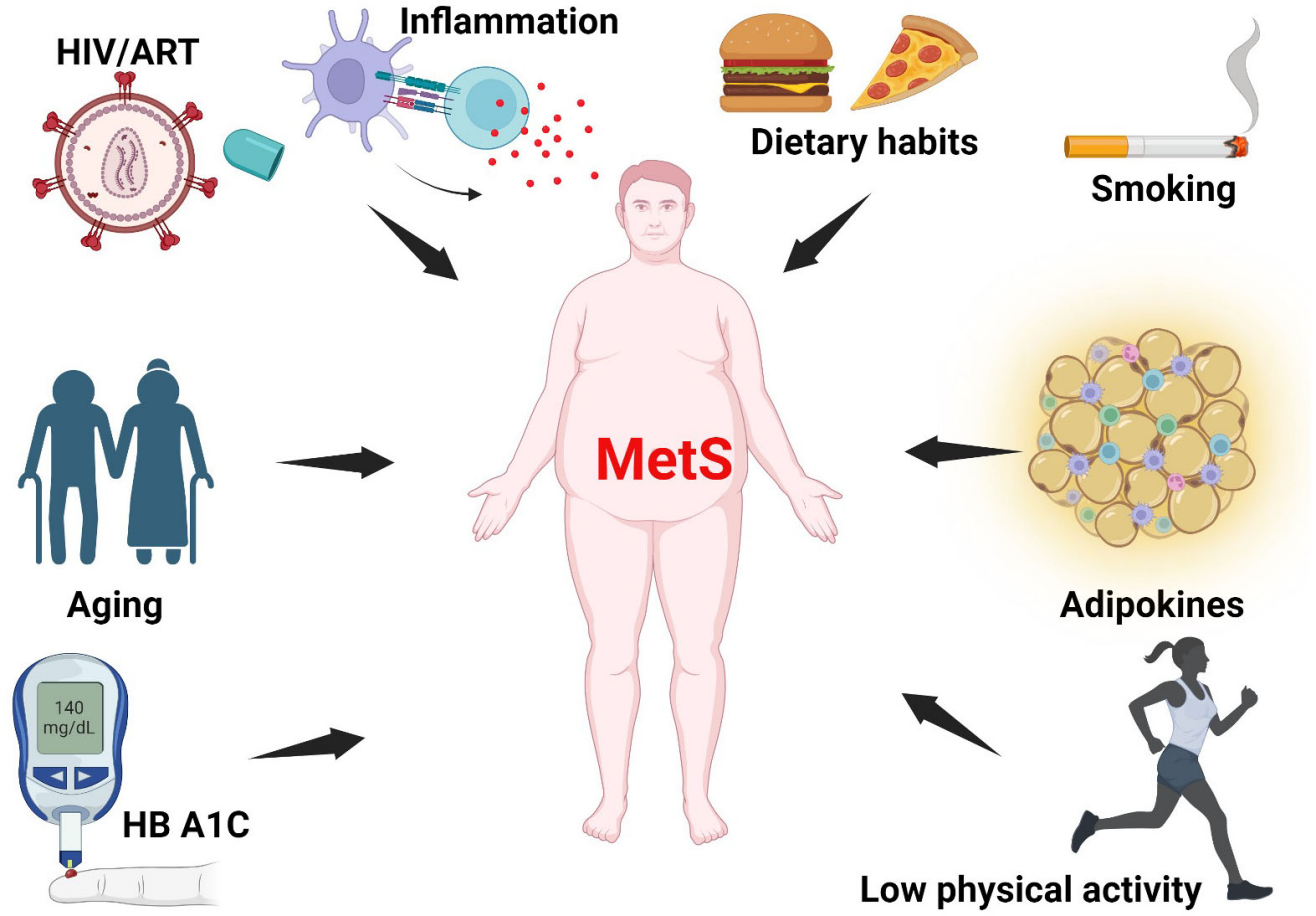
Risk factors of metabolic syndrome. MetS arises from multiple interacting factors, including chronic low-grade inflammation, poor dietary habits, smoking, impaired adipokine signaling, physical inactivity, hyperglycemia (as indicated by elevated HbA1c), aging, and HIV infection with ART. MetS, metabolic syndrome; HbA1c, glycated hemoglobin; ART, antiretroviral therapy; HIV, Human Immunodeficiency Virus. Created with BioRender.com.

In the United States (US), elevated glycated hemoglobin (HbA1c) and high total sugar consumption are associated with MetS (28). Individuals with MetS have higher values of CD45(+), CD3(+), CD4(+) T cell counts, C-reactive protein (CRP) and leptin, and lower adiponectin (23, 29). Individuals who smoke, with HIV-1, higher body mass index (BMI) (30) (31), and higher trunk-to-limb fat ratio (22) have a higher likelihood of having MetS (32), see Figure 1.

Studies have shown that ethnicity, family history, and genetic factors are key factors in an individual’s susceptibility to MetS (33). Some variants of the *APOC3* gene, which encodes apolipoprotein C3, a constituent of the triglyceride-rich lipoproteins such as very low-density lipoproteins (VLDL) are associated with increased risk of MetS (34). Lifestyle choices such as a sedentary lifestyle, also significantly contribute to the development and progression of the MetS. Lack of physical activity leads to weight gain, resulting in central obesity, which is a core component of MetS (35). Diet is another important factor as diets high in processed foods, sugars, and unhealthy fats promote IR and dyslipidemia which are key components of MetS (36). Smoking and excessive alcohol consumption are lifestyle choices that exacerbate MetS risk by contributing to IR, hypertension, and dyslipidemia (14).

Chronic inflammatory diseases, such as Chagas disease, HIV, and various autoimmune diseases, also heighten the risk of MetS. These conditions often induce systemic inflammation, which interferes with insulin signaling and lipid metabolism (12, 37). Additionally, advancing age increases the risk of MetS, as metabolic processes naturally slow down and the likelihood of developing IR increases (38). Finally, poor sleep patterns, chronic stress, and exposure to occupational or industrial noise have also been shown to be risk factors of MetS; they affect cortisol levels, insulin sensitivity, and appetite regulation, promoting weight gain and metabolic disturbances (39, 40).

## 4 Clinical features of metabolic syndrome

Metabolic syndrome is characterized by a combination of clinical features that significantly increase the risk of CVD and T2DM. Central obesity, characterized by excessive fat around the abdomen is a key feature and is normally assessed by waist circumference (41). IR, with consequent elevated blood glucose levels, is another core component, detected through fasting blood glucose or glucose tolerance tests (42). Dyslipidemia, marked by high triglycerides and low high-density lipoprotein (HDL) cholesterol, is also common, along with elevated low-density lipoprotein (LDL) cholesterol (43). Often secondary to the other components; obesity, IR, and dyslipidemia, hypertension, characterized by elevated systolic and/or diastolic blood pressure, is usually present (44). Finally, due to the synergistic action of the components of MetS, individuals with MetS have pro-inflammatory and pro-thrombotic states, which can be identified by elevated serum C-reactive protein (CRP) and fibrinogen levels, contributing to cardiovascular risk (16).

### 4.1 Metabolic syndrome diagnostic criteria

Currently, there is no consensus by medical bodies on the use of one definition and standardized diagnosis for MetS and this has resulted in various definitions and diagnostic criteria for MetS. Because of this, some components of MetS may not be acceptable by some bodies, whilst being recognized by others, leading to inconsistency in diagnosis (45).

The World Health Organization (WHO) diagnostic criteria for MetS includes presence of glucose intolerance, impaired glucose tolerance (IGT) or diabetes mellitus (DM), and/or IR, together with two or more of the components among the following: raised arterial pressure, i.e., ≥140/90 mm Hg, raised plasma triglyceride (≥150 mg/dL) and/or low HDL-C (<35 mg/dl in men and <39 mg/dl in women), central obesity, i.e., waist-hip ratio (WHR) > 0.9 in men and >0.85 in women and/or body mass index (BMI) > 30 kg/m^2^, and microalbuminuria, i.e., urinary albumin excretion rate ≥ 20 μgm/min or albumin/creatine ratio ≥ 30 μgm/mg (46).

For the European Group for Study of Insulin Resistance (EGIR) a diagnosis of MetS is made when there is elevated plasma insulin (>75th percentile) plus two other factors from among the following: Abdominal obesity: waist circumference (WC) ≥ 94 cm in men and ≥80 cm in women, hypertension: ≥140/90 mm Hg or on antihypertensive treatment, elevated triglycerides (≥150 mg/dL) and/or reduced HDL-C (<39 mg/dl for both men and women), elevated plasma glucose: impaired fasting glucose (IFG) or IGT, but no diabetes (47).

On the other hand, according to the International Federation for Diabetes (IDF), MetS diagnosis is made when the patient presents with central obesity (according to predefined standard race and gender-specific waist circumference cut-offs) plus any two of the following four parameters: raised triglycerides: ≥150 mg/dL or history of specific treatment for this lipid abnormality, reduced HDL cholesterol: <40 mg/dl in males and <50 mg/dl in females or history of specific treatment for this lipid abnormality, raised blood pressure: systolic BP ≥ 130 mm Hg or diastolic BP ≥ 85 mm Hg or on treatment for previously diagnosed hypertension, raised fasting plasma glucose: ≥100 mg/dl or previously diagnosed type 2 diabetes mellitus (48). While the harmonized criteria as one having three or more of the following characteristics; elevated waist circumference (WC, ≥94 cm for men, ≥80 cm for women), elevated fasting blood glucose (FBG, ≥5.6 mmol/L), low level of high-density lipoprotein cholesterol (HDL-c, <1.0 mmol/L for men, <1.3 for women), elevated blood pressure (BP) (systolic BP ≥ 130 or diastolic BP ≥ 85 mm Hg), and elevated triglycerides (TG, ≥1.7 mmol/L) (49).

## 5 Pathophysiology and mechanisms of metabolic syndrome

**FIGURE 2:**
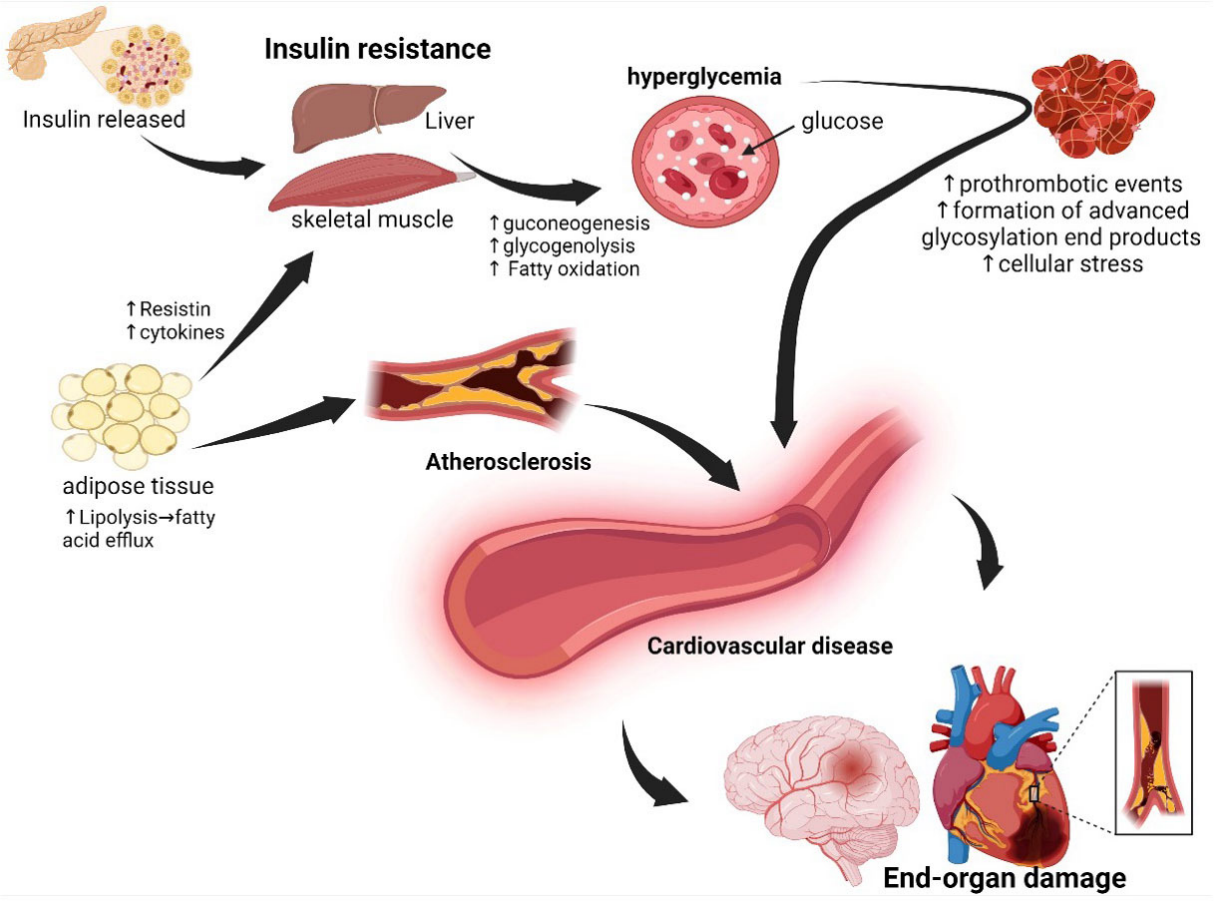
Underlying pathophysiology of metabolic syndrome: In the pathophysiology of metabolic syndrome, the cells of the liver, adipose tissue, and skeletal muscle become resistant to insulin, resulting in persistent hyperglycemia and consequent development of atherosclerosis and prothrombotic events. Adipose tissue dysfunction, atherosclerosis, oxidative stress, and prothrombotic events collectively result in endothelial dysfunction with consequent end-organ damage. Created with BioRender.com.

The mechanisms by which MetS promotes CVD are complex and involve several factors. Previous evidence has shown that MetS induces CVD through its various components. When skeletal muscle and liver cells become resistant to insulin, uptake of glucose is reduced leading to increased lipolysis in adipose tissue that contributes to atherosclerosis. Moreover, high levels of glucose damage the blood vessels via increased formation of advanced glycosylation end products and lead to other MetS conditions such as obesity, dyslipidemia, hypertension, prothrombotic state, and endothelial dysfunction, thus increasing the risk for development of cardiovascular disease (50, 51), see Figure 2.

### 5.1 Obesity and adipose tissue dysfunction

**FIGURE 3:**
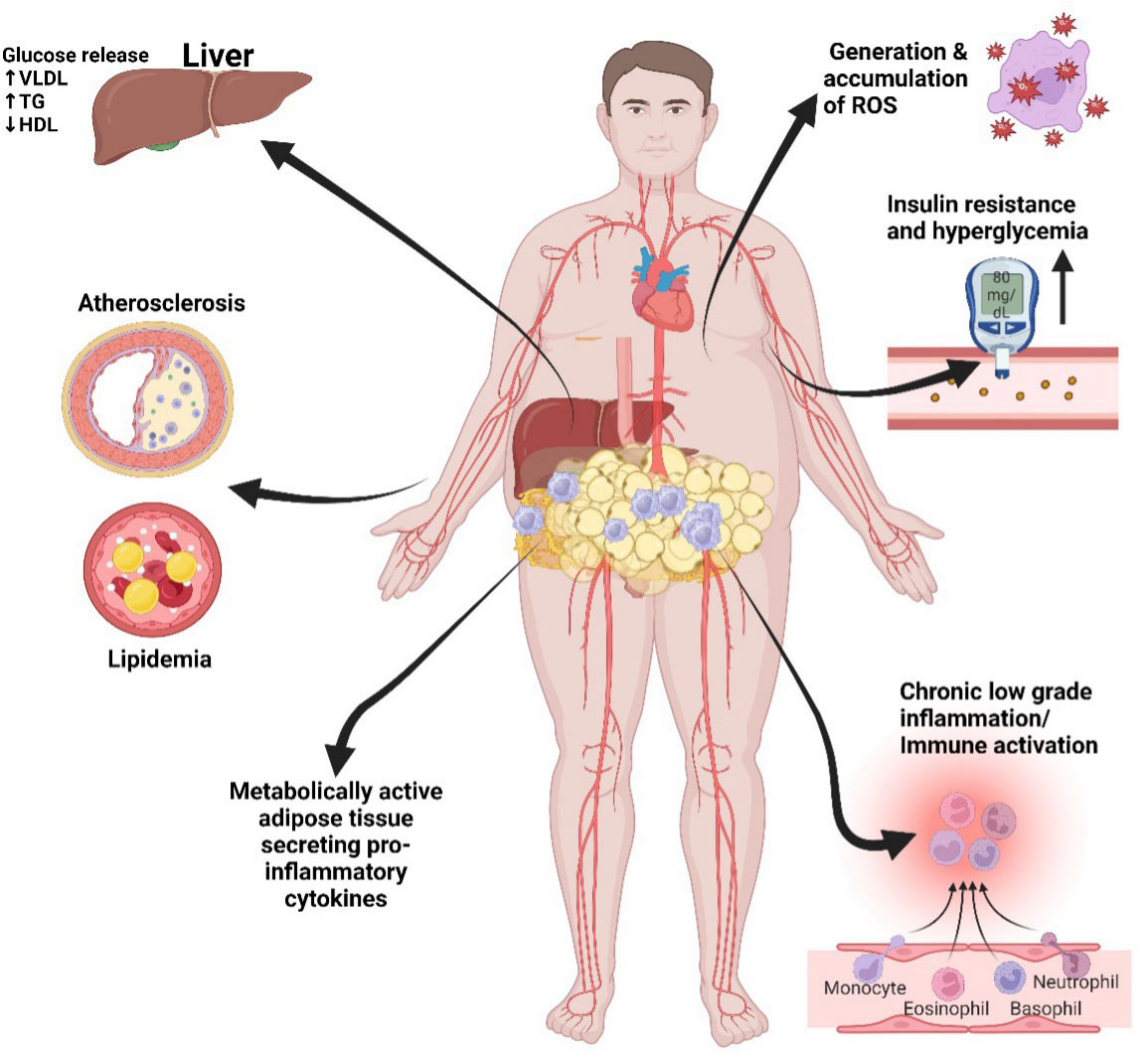
Metabolic syndrome mechanisms that promote cardiovascular disease. Visceral adipose tissue: rich in pro-inflammatory cytokines, contributes to chronic inflammation, insulin resistance, atherosclerosis, endothelial dysfunction, dyslipidemia, insulin resistance, and ultimately, the risk of CVD. LDL, low-density lipoprotein; HDL, high-density lipoprotein; TG, triglycerides; VLDL, very low-density lipoprotein; ROS, reactive oxygen species. Created with BioRender.com.

Central obesity, particularly the excessive accumulation of fat around the abdomen, known as visceral fat, is a major driver of metabolic disturbances (52, 53). This visceral fat differs from the subcutaneous fat found beneath the skin and is located around critical organs within the abdominal cavity, including the liver, pancreas, and intestines (54, 55). Its proximity to these vital organs makes visceral fat highly metabolically active and especially detrimental to health (56, 57). In obesity, adipose tissue undergoes significant structural and biochemical changes contributing to metabolic dysfunction (58). The adipocytes enlarge and proliferate, leading to an expansion of adipose tissue mass (59). This hypertrophic growth is associated with adipocyte dysfunction, characterized by altered adipokine secretion and disrupted lipid metabolism (60). Biochemically, obesity triggers chronic low-grade inflammation within adipose tissue, with increased levels of pro-inflammatory cytokines (61). This inflammation is exacerbated by the infiltration of immune cells, particularly macrophages, into adipose tissue (62). These structural and biochemical alterations collectively contribute to IR, dyslipidemia, and a pro-inflammatory state, all of which increase the risk of developing metabolic disorders, including type 2 diabetes and CVD (63), refer to Figure 3 below.

Visceral fat is not only a storage depot but also acts as an endocrine organ, releasing a variety of pro-inflammatory cytokines such as tumor necrosis factor-alpha (TNF-α), interleukin-6 (IL-6), and interleukin-1 beta (IL-1β) (64). These cytokines are potent signaling molecules that trigger inflammation throughout the body. Furthermore, adipose tissue, particularly visceral fat plays diverse roles in metabolism and inflammation. Among these, two prominent adipokines are leptin and adiponectin.

Leptin, often referred to as the “satiety hormone,” is a pro-inflammatory adipokine that plays a crucial role in regulating appetite, energy balance, and metabolism (65). However, in cases of obesity, the development of leptin resistance occurs: this is thought to be due to impaired intracellular signal transduction and reduced leptin transportation to the brain via the blood-brain barrier, rendering the body less responsive to its signals (66). In leptin resistance, there is a corresponding increase in serum leptin concentration thus leptin resistance is associated with chronic low-grade inflammation (67).

In contrast to leptin, adiponectin is generally considered to have insulin-sensitizing, anti-inflammatory, and cardioprotective properties (68, 69). Adiponectin enhances insulin sensitivity through promotion of glucose uptake and utilization in muscle and liver cells (70). It achieves this by increasing the expression and translocation of glucose transporter proteins (GLUT4) in muscle cells and hepatocytes (71, 72). Additionally, adiponectin activates AMP-activated protein kinase (AMPK), a key regulator of cellular energy balance. AMPK activation increases glucose uptake and fatty acid oxidation while inhibiting hepatic glucose production (73). The anti-inflammatory activity of adiponectin is demonstrated through its ability to suppress the production of pro-inflammatory cytokines, TNF-α and IL-6 (74, 75). Adiponectin is a potent inhibitor of nuclear factor-kappa B (NF-κB) activation, a transcription factor that regulates the expression of many pro-inflammatory genes and stimulates the production of anti-inflammatory molecules like IL-10 and interleukin-1 receptor antagonist (IL-1Ra) (76, 77). Adiponectin is thought to protect the cardiovascular system by several mechanisms. It inhibits endothelial dysfunction by increasing the production of nitric oxide (NO) and reducing oxidative stress, which helps maintain blood vessel flexibility and reduces atherosclerosis (78). Furthermore, it suppresses the adhesion of monocytes and macrophages to vascular endothelial cells, reducing the inflammatory response in blood vessels (79, 80). Adiponectin has anti-thrombotic properties by inhibiting platelet activation and aggregation, reducing the risk of blood clots. It also plays a role in reducing cardiac hypertrophy and fibrosis, which are common factors in heart failure (81). Obesity, particularly central obesity, is linked to lower adiponectin levels. Reduced adiponectin levels are closely associated with IR and an elevated risk of CVD. The molecular mechanisms behind this association involve the dysfunction of adipose tissue, primarily due to inflammation and IR (82). The enlarged visceral adipocytes release pro-inflammatory cytokines which suppress adiponectin production at the molecular level (83). Moreover, localized hypoxia within the expanded visceral fat prompts the release of hypoxia-inducible factors that further hinder adiponectin gene expression and secretion (84). Consequently, lower adiponectin levels contribute to metabolic dysfunction and increased risk of MetS and cardiovascular disease (85).

The pro-inflammatory cytokines and adipokines released by visceral fat contribute significantly to systemic inflammation. This inflammation disrupts the body’s insulin signaling pathways, exacerbating IR (86). Moreover, the inflammatory mediators released by visceral fat can also directly impact blood vessels, contributing to endothelial dysfunction, characterized by impaired dilation and constriction of blood vessels (55, 87, 88). This dysfunction serves as a precursor to hypertension (high blood pressure), which, in turn, is a significant risk factor for CVD (89, 90). Additionally, the inflammatory processes triggered by central obesity can influence lipid metabolism, leading to dyslipidemia (58).

### 5.2 Dyslipidemia

Dyslipidemia in MetS is characterized by elevated triglycerides, reduced HDL cholesterol, and increased levels of small, dense low-density lipoprotein (LDL) particles (91, 92). Dyslipidemia promotes atherosclerosis through multiple mechanisms. Elevated triglycerides are associated with increased very low-density lipoprotein (VLDL) production by the liver, contributing to the formation of atherogenic lipoproteins (93). Reduced HDL cholesterol impairs reverse cholesterol transport, hindering the removal of excess cholesterol from peripheral tissues (94). LDL, especially small, dense LDL particles are more prone to oxidation, further exacerbating endothelial damage (95).

Atherosclerosis begins with the deposition of oxidized LDL particles in the arterial walls (96). The oxidative modification of LDL makes it more adhesive and readily taken up by macrophages, resulting in the formation of foam cells (97). As these lipid-laden foam cells accumulate within the arterial walls, they trigger a localized immune response and the release of pro-inflammatory cytokines. These events lead to the recruitment of more immune cells, particularly macrophages, to the site of injury (98). Macrophages, in an attempt to clear the accumulating lipids, become engorged, further contributing to the formation of fatty streaks and plaque development (99). As the plaques grow over time, they can protrude into the arterial lumen, narrowing the vessel and potentially obstructing blood flow (100).

Moreover, the inflammatory pathways activated within the arterial walls stimulate the release of growth factors, which encourage the proliferation of smooth muscle cells (101). The proliferation of these cells leads to the thickening of the arterial walls and the formation of a fibrous cap over the lipid-rich core of the plaque (102). This cap is prone to rupture, which is a critical event in the development of acute cardiovascular events such as myocardial infarction and stroke (103).

Dyslipidemia in MetS therefore fuels the molecular mechanisms underlying atherosclerosis and the progression of CVD (104). Elevated triglycerides, reduced HDL cholesterol, and small, dense LDL particles create an environment that fosters the formation of atherosclerotic plaques through increased VLDL production, impaired reverse cholesterol transport, and heightened oxidative stress (105). These processes ultimately lead to inflammation, foam cell formation, plaque growth, and potential plaque instability, all of which increase the risk of cardiovascular events (106).

### 5.3 Hypertension

Hypertension, is one of the most prevalent components of MetS, and exerts significant deleterious effects on blood vessels through the activation of the Renin-Angiotensin System (RAS), ultimately promoting the development and progression of atherosclerosis as previously discussed (107, 108). This complex molecular mechanism begins within adipose tissue, where adipocytes secrete angiotensinogen, an inactive precursor protein produced primarily by the liver, creating a crucial link between obesity and MetS (109, 110). Once secreted, angiotensinogen undergoes enzymatic cleavage by renin, which is primarily produced by the kidneys in response to RAS activation, into angiotensin I (Ang I), with any disruption in this balance contributing to hypertension (111). Ang I is subsequently converted into Angiotensin II (Ang II) through the action of angiotensin-converting enzyme (ACE), primarily found in the lungs. Ang II, as the central effector molecule of the RAS, induces vasoconstriction, sodium retention, and aldosterone release, collectively increasing blood pressure (112–114).

The persistent elevation of Ang II levels in hypertension leads to endothelial dysfunction, characterized by oxidative stress, inflammation, and pro-inflammatory cytokine production, impairing endothelial function and its integrity (115). This dysfunction, in turn, promotes atherosclerosis as the damaged endothelium allows LDL cholesterol to infiltrate the arterial wall, initiating plaque formation. High blood pressure further contributes to atherosclerosis by placing increased stress on blood vessel walls, promoting inflammation, and damaging the endothelium (116, 117). Additionally, obesity-related pro-inflammatory molecules released by adipose tissue exacerbate inflammation, ultimately contributing to endothelial dysfunction, plaque formation, and the progression of atherosclerosis (2, 118, 119).

### 5.4 Oxidative stress

**FIGURE 4:**
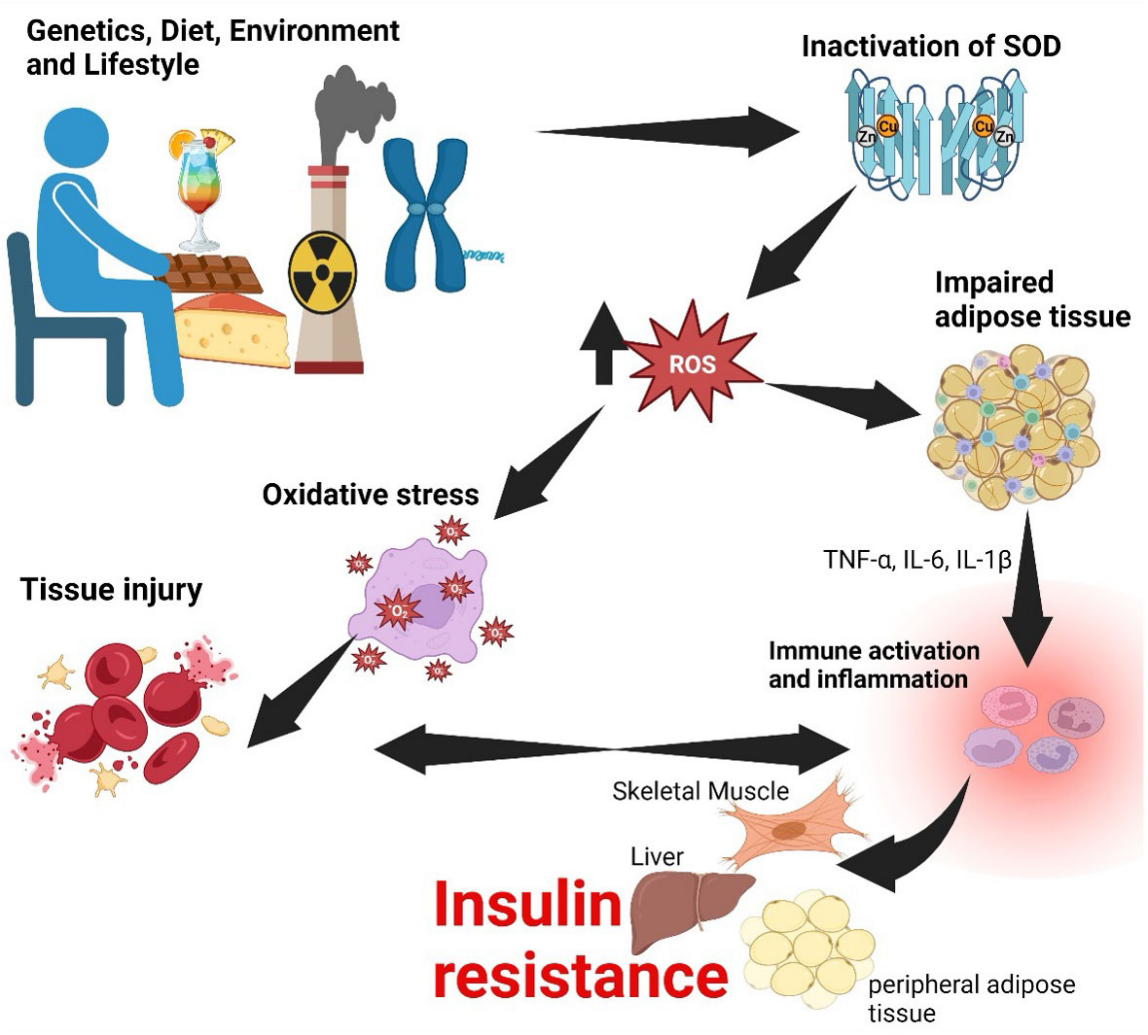
Relationship between oxidative stress, inflammation, and insulin resistance. Genetic, dietary, environmental and lifestyle factors lead to inactivation of SOD-1 and SOD-2. Inactivation of SOD enzymes has a consequence of an increase in ROS causing oxidative stress, which injures tissue. Injury to tissue and pro-inflammatory cytokines secreted by impaired adipose tissue culminate in inflammation. If this process becomes persistent, inflammation becomes chronic and may have a consequence of insulin resistance. SOD, superoxide dismutase; ROS, reactive oxygen species; TNF, tumor necrosis factor; IL, interleukin. Created with BioRender.com.

Oxidative stress occurs when there is an imbalance between the production of reactive oxygen species (free radicals) and the body’s ability to neutralize them with antioxidants (120). Superoxide dismutase (SOD) enzymes, such as SOD 1 in the cytoplasm and SOD 2 in the mitochondria, are crucial proteins that neutralize superoxide radicals (O^2^) (121). In MetS, these enzymes may become inactivated through several mechanisms (Figure 4) (122). High blood sugar levels and dyslipidemia can lead to glycation and lipid peroxidation, modifying SOD enzymes and rendering them less effective in neutralizing superoxide radicals (123). Additionally, impaired mitochondrial function in MetS can affect the activity of SOD2, as it operates within the mitochondria (124). Mitochondrial dysfunction leads to increased superoxide production and diminished antioxidant capacity (125).

The progression of CVD in MetS due to oxidative stress is multifaceted. Oxidative stress damages the endothelial lining of blood vessels, leading to endothelial dysfunction, vasoconstriction, inflammation, and atherosclerosis (126). ROS promote the oxidation of LDL cholesterol, making it more atherogenic, and oxidized LDL is taken up by macrophages, resulting in the formation of foam cells within atherosclerotic plaques (127). Furthermore, oxidative stress triggers inflammatory pathways, accelerating atherosclerosis and creating a vicious cycle of oxidative stress and inflammation (128). The unstable fibrous cap of atherosclerotic plaques, weakened by oxidative stress, is vulnerable to rupture, potentially leading to acute cardiovascular events like heart attacks and strokes (129). Ultimately, oxidative stress in MetS is a critical contributor to the development and progression of CVD, and it can result from the inactivation of SOD1 and SOD2 (130). This cascade of events, including endothelial dysfunction, atherosclerosis, inflammation, and plaque rupture, increases the risk of CVD, emphasizing the importance of managing oxidative stress and maintaining antioxidant defenses in individuals with MetS (131).

### 5.5 Insulin resistance

As previously discussed, IR lies at the heart of MetS, a constellation of risk factors that significantly elevate the chances of developing type 2 diabetes and cardiovascular disease. The mechanisms behind IR are interconnected, involving genetic, environmental, inflammatory, oxidative, and metabolic factors (122). Among the primary pathways contributing to IR, abnormalities in the insulin receptor and its downstream signaling molecules take center stage. Any defects in these critical components can disrupt the cascade of events initiated by insulin, impairing glucose uptake and utilization in various tissues (132, 133).

Additionally, lipotoxicity and ectopic fat accumulation play a significant role, particularly in the context of obesity (134). Excess visceral fat releases free fatty acids into the bloodstream, interfering with insulin signaling and leading to the accumulation of harmful lipid metabolites in tissues like the liver, muscle, and heart. This lipotoxic environment activates kinases like protein kinase C (PKC), c-Jun N-terminal Kinase (JNK), and Inhibitory kappa B kinase beta (IKKβ), which in turn, phosphorylate insulin receptor substrates, hindering their function (135).

Chronic low-grade inflammation further exacerbates IR. Obesity and MetS are associated with elevated levels of pro-inflammatory cytokines, which can activate the same kinases as lipotoxicity and promote the degradation of insulin receptor substrates (136). Oxidative stress, characterized by an imbalance between ROS production and antioxidant defenses, also contributes to IR. ROS can damage cellular components and activate kinases, ultimately impairing insulin signaling (137).

### 5.6 Chronic inflammation

As noted earlier, most pathological processes associated with MetS result in activation of low-grade inflammation. Chronic low-grade inflammation plays a central role in the development and progression of CVD through a complex interplay of molecular mechanisms (138). At the core of this process lies chronic activation of the immune system, primarily orchestrated by pro-inflammatory cytokines such as IL-6, TNF-α, and IL-1β (139). These cytokines promote the recruitment of immune cells, particularly monocytes, to the vascular endothelium (140). Once recruited, monocytes differentiate into macrophages and subsequently engulf oxidized LDL particles, giving rise to foam cells, a hallmark of atherosclerosis (98). These foam cells accumulate within arterial walls, leading to the formation of atherosclerotic plaques (141).

Chronic inflammation also exacerbates plaque instability. Macrophages within the plaque produce matrix metalloproteinases (MMPs), which weaken the fibrous cap of the plaque, making it susceptible to rupture (142). Ruptured plaques can trigger thrombosis, leading to myocardial infarction or stroke (143). Furthermore, the ongoing inflammatory milieu induces endothelial dysfunction, reducing the production of vasodilatory nitric oxide (NO) and increasing the release of endothelin-1 (ET-1), promoting vasoconstriction and hypertension (144). Moreover, inflammation stimulates the liver to release acute-phase proteins like c-reactive protein (CRP), which are elevated in CVD patients and serve as clinical markers of inflammation (145). Elevated CRP levels are associated with increased CVD risk, and CRP itself may directly contribute to endothelial dysfunction and plaque instability (146). Finally, the continuous inflammatory response induces a state of IR, impairing glucose metabolism, and promoting dyslipidemia, two factors that amplify CVD risk (147).

## 6 Complications of metabolic syndrome

Metabolic syndrome increases the odds of developing CVD such as coronary artery disease, stroke, and peripheral artery disease. The presence of hypertension, dyslipidemia, and central obesity in MetS has a synergistic effect on CVD risk (148). Hypertension is associated with hypertrophy and eventual heart failure (149). Dyslipidemia, which has a signature rise in triglycerides and low HDL cholesterol, leads to atherosclerosis which is strongly associated with myocardial infarction and stroke (150). Additionally, the pro-inflammatory and pro-thrombotic states associated with MetS promote blood clot formation, further increasing the CVD risk (151).

Type 2 diabetes mellitus T2DM is a common complication of MetS. Insulin resistance, a core feature of metabolic syndrome, impairs glucose uptake by cells, leading to consistently elevated blood glucose levels (152). The coexistence of insulin resistance and central obesity exacerbates this process since excess adipose tissue releases free fatty acids and inflammatory cytokines that further impair insulin signaling (153). T2DM increases the risk of microvascular complications, including retinopathy, nephropathy, and neuropathy, as well as macrovascular complications, such as heart disease and stroke (154).

Metabolic dysfunction-associated steatotic liver disease (MASLD), formerly known as non-alcoholic fatty liver disease (NAFLD), is increasingly recognized as the hepatic component of metabolic syndrome (MetS) (155, 156). It arises from the same core disturbances, namely central obesity, insulin resistance, and dyslipidemia and shares overlapping pathophysiology with other MetS features (157). Nutritionally, MASLD is strongly driven by poor dietary patterns, mainly the excessive intake of refined carbohydrates, saturated fats, and fructose, which promote hepatic fat accumulation and inflammation (158). The condition can progress to steatohepatitis, fibrosis, and cirrhosis, while also worsening systemic insulin resistance and lipid dysregulation (159). As such, MASLD both reflects and reinforces metabolic dysfunction. Its rising prevalence highlights the urgent need for early dietary intervention and population-level strategies targeting nutritional quality and metabolic health (160).

Another frequent complication of MetS is Polycystic ovary syndrome (PCOS) in women, it is characterized by irregular menstrual cycles, hyperandrogenism, and polycystic ovaries (161). Insulin resistance plays an important role in the pathophysiology of PCOS, as high insulin levels stimulate androgen production by the ovaries, resulting in symptoms such as hirsutism, acne, and anovulation (162). Women with PCOS are at an increased risk of developing T2DM, CVD, and endometrial cancer due to chronic anovulation and unopposed estrogen exposure (163, 164).

Metabolic syndrome is associated with an increased risk of developing chronic kidney disease (CKD). Hypertension and hyperglycemia which are common features of MetS, contribute to kidney damage by causing glomerular hyperfiltration and promoting the development of diabetic nephropathy (165). Dyslipidemia and the pro-inflammatory state of MetS further increase renal injury. CKD can progress to end-stage renal disease (ESRD), which requires dialysis or kidney transplantation (166).

## 7 Metabolic syndrome management and therapeutic approaches

Efficacious treatment and effective prevention of MetS require that multitudinous risk factors be addressed simultaneously to attain a significant reduction in morbidity and mortality globally. Therefore, it is imperative that individuals with MetS and/or any component of MetS are identified to offer evidence-based curative/preventive medicine. The current therapeutic approaches for MetS are aimed at managing individual risk factors. These approaches include:

### 7.1 Physical activity

Lifestyle modification plays an important role in the comprehensive treatment and effective management of MetS and is a pillar of a successful strategy to mitigate the adverse effects of MetS and improve overall health. Physical activities play an important role in reducing fat in individuals with MetS, enhancing fat metabolism, and improving overall metabolic health (167).

#### 7.1.1 Aerobic exercise

Aerobic exercise that can include activities like brisk walking, swimming, running, or cycling taps into the body’s fat stores for energy, releasing fatty acids through lipolysis, a crucial step in treating MetS, by reducing fat accumulation and improving insulin sensitivity (168). Such exercises also stimulate mitochondrial biogenesis, coordinating various signaling pathways to optimize energy production and enhance endurance capacities (169). Additionally, it promotes glycogen storage by increasing glucose uptake through insulin signaling and AMPK activation (170). This stored glycogen supports energy demands, regulates blood glucose levels, and prevents IR, contributing to a holistic approach to metabolic health (171). An example of aerobic exercise is Moderate-Intensity Continuous Training (MICT).

Moderate-Intensity Continuous Training exercise offers substantial benefits in the management of MetS (172). Engaging in sustained, moderate-intensity activities like brisk walking, cycling, or swimming regularly improves insulin sensitivity, glucose regulation, and lipid profiles. MICT enhances cardiovascular health, reducing the risk factors associated with MetS, such as hypertension and dyslipidemia (173). This form of exercise also aids in weight management by promoting fat utilization for energy (174). Regular MICT enhances insulin sensitivity by promoting the translocation of glucose transporter type 4 (GLUT4), facilitating increased glucose uptake by skeletal muscles (175). This process is mediated by the activation of AMPK and improved mitochondrial function, optimizing cellular energy metabolism (176).

#### 7.1.2 High-intensity interval training

High-Intensity Interval Training (HIIT) is a type of exercise that is considered to have both aspects of aerobic and anaerobic exercise (177). It is utilized in treating MetS by incorporating short bursts of intense exercise alternated with periods of rest or lower-intensity activity (178). While there is no one-size-fits-all prescription, a common approach involves 20–30 s of vigorous exercise, such as sprinting or cycling, followed by 30 s to a minute of rest or low-intensity activity (177). This cycle is typically repeated for about 20–30 min. HIIT has shown effectiveness in improving insulin sensitivity, glucose regulation, and lipid profiles, making it a valuable component in managing MetS. The adaptability of HIIT allows for customization based on individual fitness levels, making it accessible and time-efficient for a wide range of individuals (177). HIIT enhances insulin sensitivity by promoting glucose uptake and utilization in skeletal muscles, driven by increased AMPK activity. The intense bursts of exercise induce favorable changes in muscle fiber composition, favoring insulin-sensitive fibers. HIIT also stimulates the release of myokines, contributing to anti-inflammatory effects and improved insulin sensitivity (179). The short, intense bouts of exercise trigger mitochondrial biogenesis through the activation of peroxisome proliferator-activated receptor-gamma coactivator 1-alpha (PGC-1α), enhancing oxidative metabolism (180). Furthermore, HIIT induces favorable changes in adipokine secretion, promoting metabolic health. These molecular adaptations collectively establish HIIT as a potent intervention, addressing IR, inflammation, and mitochondrial function, and providing a multifaceted approach to MetS treatment (181).

#### 7.1.3 Strength training

Strength training, a type of anaerobic exercise, is also beneficial in the treatment of MetS by promoting muscle mass development and activating pathways that enhance metabolic health (182). Strength training regimen involves performing compound exercises like squats, deadlifts, and bench presses, progressively increasing resistance (load) (183). This process stimulates muscle hypertrophy, improving insulin sensitivity, glucose metabolism, and lipid profiles. The overall resulting mechanism of strength training on cellular metabolic activity is similar to that of HIIT (181, 184). In essence, strength training’s molecular adaptations offer a comprehensive strategy, not only addressing IR and inflammation but also harnessing the principle of a heightened resting metabolic rate for effective MetS treatment (185). A well-rounded program, incorporating both resistance and aerobic exercises, is often recommended (186).

#### 7.1.4 Lifestyle activity

Non-Exercise Activity Thermogenesis (NEAT) is a lifestyle strategy for treating MetS by increasing daily energy expenditure. Without a prescribed regimen, the focus is on seamlessly integrating physical activity into daily routines, like choosing stairs or engaging in short walks (187). NEAT aligns with a holistic lifestyle modification plan, emphasizing a balanced diet and stress management. The approach, supported by healthcare professionals, promotes metabolic health by breaking up prolonged sitting and encouraging active transportation (188). Simultaneously, incorporating various exercise modalities, including aerobic, resistance, and lifestyle activities, reduces fat, enhances mitochondrial biogenesis, and improves insulin sensitivity (189).

### 7.2 Diet as therapy for metabolic syndrome

A balanced diet plays a central role in reducing fat in individuals with MetS, affecting various molecular mechanisms within the body (190). These mechanisms collectively contribute to fat reduction and overall metabolic health in several ways (191).

#### 7.2.1 Benefits of a balanced diet low in fat

A well-balanced diet, low in refined carbohydrates and added sugars, enhances insulin sensitivity through several mechanisms. Primarily, it reduces the rapid influx of glucose into the bloodstream, preventing excessive insulin secretion by the pancreas (192). This lower demand for insulin helps maintain the sensitivity of insulin receptors on target cells (193). Additionally, a diet low in refined carbohydrates and added sugars decreases the formation of advanced glycation end-products (AGEs) and mitigates oxidative stress, both of which contribute to IR (194). Reducing refined carbohydrates and added sugars also lowers the release of inflammatory cytokines, thus diminishing chronic inflammation, which is closely linked to IR (195). Furthermore, this dietary approach supports the balanced production of hormones like adiponectin, which improves insulin action (196).

#### 7.2.2 Macronutrients in diet, appetite balance and calorie loss

A diet with the correct proportions of macronutrients plays a central role in regulating the balance of appetite-regulating hormones, chiefly ghrelin and leptin (197). Ghrelin stimulates appetite when its levels are elevated (198). As earlier discussed, leptin signals fullness and inhibits hunger. When an individual consumes a diet that has the right balance between proteins, fats, and carbohydrates, these hormones work harmoniously. Adequate protein intake, for instance, promotes a sense of fullness by increasing the release of peptide YY and cholecystokinin which are key in appetite regulation and satiety, signaling to the brain to induce feelings of fullness and suppress hunger (199). This optimization of hormonal responses effectively controls hunger and prevents overeating, creating a caloric deficit essential for fat loss. The interplay of these hormones is central to appetite regulation, making a balanced diet a crucial component of successful weight management (200). Furthermore, a balanced diet, particularly one with adequate protein, increases the thermic effect of food (TEF) (201, 202). This effect refers to the energy expended during the digestion and metabolism of nutrients. Protein has a higher TEF compared to other macronutrients, meaning more calories are burned during the digestion and absorption of protein-rich foods (203).

#### 7.2.3 Adequate protein, sustained muscle synthesis and increased resting metabolic rate

A well-balanced diet with adequate protein intake plays an important role in the preservation of lean muscle mass during weight loss (204). Since proteins consist of amino acids, they serve as the fundamental building blocks of muscle tissue. Consuming an adequate amount of protein triggers signals that encourage the body to sustain muscle protein synthesis, even as calorie intake decreases (205). This muscle preservation is of paramount importance since muscle tissue is metabolically active, necessitating more energy for maintenance compared to fat (206). Consequently, individuals who retain their lean muscle mass enjoy a higher resting metabolic rate, which results in the burning of more calories during periods of rest (207, 208).

#### 7.2.4 Essential fatty acids and lipid metabolism

In MetS, a balanced diet assumes a critical role as the adequate intake of essential fatty acids, including omega-3 and omega-6 fatty acids, promotes overall health (209). These fatty acids are indispensable for various metabolic processes, particularly the oxidation of fats. Omega-3 fatty acids, in particular, exert a pronounced influence by modulating gene expression involved in lipid metabolism, enhancing the breakdown of stored fat for energy (210). Their role extends toward mitigation of inflammation, thus ameliorating its suppressive effect on lipid metabolism (211). Furthermore, omega-3 fatty acids promote mitochondrial biogenesis, augmenting cellular capacity for efficient fatty acid utilization (212). Thus a balanced diet encompassing essential fatty acids is key in creating a metabolic environment conducive to effective lipolysis and ultimately contributing to fat reduction in MetS (213).

#### 7.2.5 Fruits and vegetables, and optimized mitochondrial efficiency

A diet that includes nutrient-rich foods, such as fruits and vegetables, supports mitochondrial function (214). This dietary approach contributes to enhanced mitochondrial biogenesis within muscle cells, amplifying their capacity to oxidize stored fat for energy (215). The micronutrients and antioxidants present in fruits and vegetables play a pivotal role in optimizing mitochondrial efficiency (216). Furthermore, the activation of signaling pathways, such as AMPK and PGC-1α, are stimulated by these nutrient-rich foods (217, 218). This culminates in an augmented mitochondrial network, enhancing the cell’s ability to catabolize fatty acids and promoting an environment conducive to effective fat utilization, a key aspect of fat reduction in MetS (219).

#### 7.2.6 Diet and pro-fat metabolism gene expression

A balanced diet holds the capacity to exert influence on epigenetic modifications governing gene expression pertinent to fat metabolism (220). Certain compounds inherent in dietary components, such as resveratrol present in grapes and red wine, possess the ability to modulate gene expression patterns conducive to fat reduction (221). Resveratrol, a polyphenol, engages with key molecular pathways involved in epigenetic regulation, influencing histone modification and DNA methylation (222). This interaction triggers alterations in gene expression, favoring mechanisms that enhance fat metabolism (223). The interplay between dietary compounds and epigenetic modifications highlights the potential of a balanced diet, enriched with specific bioactive components, in shaping the molecular landscape conducive to effective fat reduction (224).

#### 7.2.7 Benefit of high dietary fiber

High-fiber foods exert an important influence on fat loss in MetS through several mechanisms. Primarily, soluble fiber undergoes fermentation by gut bacteria, producing short-chain fatty acids (SCFAs) (225). These SCFAs contribute to appetite regulation and energy metabolism (226). SCFAs contribute to appetite regulation by binding to receptors on gut cells, triggering the release of satiety-inducing hormones like peptide YY and glucagon-like peptide-1 (227). Furthermore, fiber slows the digestion and absorption of carbohydrates, stabilizing blood sugar levels and reducing insulin spikes that may contribute to fat accumulation (228). Additionally, the fermentation process influences the gut microbiota composition, promoting the growth of beneficial bacteria associated with improved metabolism (229). High-fiber foods also contribute to enhanced satiety, reducing overall calorie intake (230). Collectively, these mechanisms showcase how incorporating high-fiber foods into the diet can positively impact fat loss in individuals with MetS.

#### 7.2.8 Hydration

Hydration plays a crucial role in promoting fat loss in MetS through several ways. Firstly, adequate water intake enhances lipolysis, the breakdown of stored fat, by supporting hydrolytic enzymatic activities involved in fat mobilization (231). Proper hydration also aids in thermogenesis, the process by which the body generates heat and burns calories, contributing to increased energy expenditure (232). Hydration improves thermogenesis by optimizing cellular processes. Water is essential for the function of mitochondria thus adequate hydration supports efficient oxidative phosphorylation, ensuring optimal energy production and heat generation, thereby contributing to increased thermogenic activity in the body (233). Additionally, water is essential for optimal kidney function, facilitating the elimination of waste products, including by-products of fat metabolism (234). Moreover, hydration supports cellular processes that utilize stored fat for energy, promoting an environment conducive to fat loss (235).

#### 7.2.9 Fasting-mimicking diets

Fasting-mimicking diets (FMDs) work by inducing physiological changes like those observed during periods of fasting. These diets, typically low in calories and macronutrients, trigger cellular responses that promote metabolic health (236). FMDs influence key pathways such as AMPK and sirtuins, enhancing cellular stress resistance and promoting mitochondrial function (237, 238). Moreover, they stimulate autophagy, a cellular recycling process that eliminates damaged components, and reduce inflammation (238). These molecular adaptations collectively contribute to improved insulin sensitivity, glucose regulation, and lipid metabolism.

#### 7.2.10 The Mediterranean diet

Among the proposed dietary interventions for MetS, dietary modification remains the most practical and sustainable approach for large-scale population impact. Among the various dietary models, the Mediterranean diet stands out because of its consistent association with metabolic and cardiovascular benefits. The Mediterranean diet emphasizes high consumption of vegetables, fruits, legumes, whole grains, nuts, and olive oil as the principal source of fat. It promotes moderate intake of fish, poultry, and dairy, while discouraging red and processed meats, trans-fats, and added sugars (213, 239).

Several studies have shown that adherence to the Mediterranean diet reduces the incidence and severity of MetS. Individuals at high cardiovascular risk who adhere to the Mediterranean diet supplemented with extra virgin olive oil or mixed nuts show significant improvements in body weight, waist circumference, systolic and diastolic blood pressure, lipid profile, and fasting plasma glucose. These changes result in lower rates of type 2 T2DM, myocardial infarction, and stroke, which are all key complications linked to MetS (239–241).

Mechanistically, the Mediterranean diet achieves control of MetS through multiple biologically plausible pathways. Its high content of monounsaturated fats, principally oleic acid from olive oil, improves insulin sensitivity, reduces hepatic fat accumulation, and promotes favorable lipid metabolism (213, 242, 243). The fiber-rich and antioxidant-dense components of the diet, primarily from fruits, vegetables, legumes, and nuts, lead to reductions in oxidative stress and low-grade systemic inflammation, both of which are central to the progression of MetS (244). Additionally, some studies have shown that the Mediterranean diet modulates the gut microbiota. Increase in microbial diversity and higher abundance of short-chain fatty acid producing bacteria have been reported, which may play a role in improving glucose tolerance, reducing endotoxemia, and enhancing immune regulation (245).

Other dietary strategies, including the DASH (Dietary Approaches to Stop Hypertension) diet and whole-food, plant-based diets, also offer metabolic benefits. However, the Mediterranean diet remains the most extensively validated in both clinical and epidemiological settings, with broad applicability and high adherence rates, even outside Mediterranean populations (246, 247).

Given its numerous benefits, including its effects on metabolism, inflammation, and vascular health, the Mediterranean diet is a rational and evidence-based therapeutic option for managing MetS. When combined with physical activity, and micronutrient optimization, this dietary approach may support the reversal of early metabolic derangements, improve endothelial function, and reduce the risk of major complications such as T2DM, cardiovascular disease, and erectile dysfunction (248, 249).

### 7.3 Metabolic syndrome medication

Medication may be necessary to manage some of the risk factors for MetS. Medications that may be used include Metformin which is a widely used medication for managing high blood sugar levels, particularly in individuals with diabetes or prediabetes. It works by improving the body’s sensitivity to insulin and reducing the production of glucose by the liver. By controlling blood sugar levels, Metformin helps to lower one of the key risk factors for MetS.

Thiazolidinediones (TZDs) are antidiabetic drugs prescribed in MetS management to enhance insulin sensitivity (250). They increase insulin sensitivity by activating the peroxisome proliferator-activated receptor gamma (PPAR-γ) (251). Activation of PPAR-γ enhances glucose uptake in adipose and muscle tissue while suppressing hepatic gluconeogenesis, thus optimizing glycemic control (252). TZD also improves lipid metabolism and has anti-inflammatory properties (253).

Statins are a class of drugs prescribed in the management of high cholesterol levels, specifically the reduction of LDL cholesterol (254). High cholesterol is a common component of MetS and a risk factor for CVD. Statins work by inhibiting the enzyme 3-hydroxy-3-methylglutaryl coenzyme-A (HMG-CoA) reductase, which is involved in cholesterol production in the liver (255, 256). By slowing cholesterol production, they lower LDL cholesterol levels in the bloodstream. Additionally, statins have anti-inflammatory properties and thus retard plaque buildup in arteries, thereby reducing the risk of atherosclerosis and heart disease (257, 258). Statins are beneficial for individuals with MetS to address the lipid profile component of the syndrome (259).

Bile Acid Sequestrants are a class of drugs often used to lower LDL cholesterol levels in MetS (260). They function by binding to bile acids in the small intestine, preventing their reabsorption into the bloodstream. As a result, these complexes of bile acids and the drug remain in the intestinal tract and are eventually excreted from the body (261). As compensation for the loss of bile acids, the liver increases the conversion of cholesterol into new bile acids, reducing the pool of cholesterol in the liver (262). The liver then takes up LDL cholesterol from the bloodstream to synthesize more bile acids, leading to a decrease in LDL cholesterol levels in the blood (263). Their mode of action makes bile acid sequestrants effective in lowering LDL cholesterol and is particularly valuable in managing conditions characterized by high cholesterol levels, including MetS (264).

Niacin (Vitamin B3) can be used to increase HDL cholesterol levels and lower LDL cholesterol and triglycerides (265). It works by inhibiting the liver’s production of VLDL cholesterol, a precursor to LDL (266). Niacin also has favorable effects on lipid profiles. Prolonged niacin treatment stimulates adipose tissue to enhance the synthesis of polyunsaturated fatty acids (PUFAs), particularly omega-3 and omega-6 fatty acids. This effect is mediated through the activation of niacin receptors, leading to increased expression of desaturase enzymes involved in PUFA biosynthesis. The heightened PUFA synthesis results in an anti-inflammatory lipid and oxylipin plasma profile, influencing various inflammatory pathways. These beneficial changes, including decreased pro-inflammatory eicosanoids, contribute to the observed anti-inflammatory effects of niacin (267, 268).

Fibrates are medications used to lower triglyceride levels and increase HDL cholesterol (often referred to as “good” cholesterol). Elevated triglycerides are common in MetS, and having low levels of HDL cholesterol is a risk factor for heart disease (269). Fibrates work by activating peroxisome proliferator-activated receptors (PPARs), which regulate lipid metabolism (270). These medications can help improve the lipid profile, especially in individuals with high triglycerides (271).

Elevated blood pressure is another key aspect of MetS (272). Antihypertensive medications, including Angiotensin-Converting Enzyme (ACE) inhibitors, beta-blockers, and diuretics, are prescribed to manage hypertension. ACE Inhibitors inhibit the conversion of angiotensin I to angiotensin II, a potent vasoconstrictor (273, 274). By blocking this process, they dilate blood vessels, reducing blood pressure. They also help protect the heart and kidneys from the damaging effects of high blood pressure (275). Beta-blockers work by blocking the effects of adrenaline on the heart, thus reducing heart rate and blood pressure (276). They also help reduce the workload on the heart, making it useful for individuals with both hypertension and heart-related conditions (277). Diuretics help the body eliminate excess sodium and water, reducing the volume of blood and lowering blood pressure (278). They are often used in combination with other antihypertensive drugs to enhance their effectiveness. Lowering blood pressure helps protect the heart and blood vessels (279, 280).

### 7.4 New and emerging therapies for the treatment of metabolic syndrome

#### 7.4.1 Sodium-Glucose Co-Transporter 2 inhibitors

Sodium-Glucose Co-Transporter 2 (SGLT-2) inhibitors were recommended for the management of diabetes mellitus in 2022 and have shown great potential in the management of MetS (281, 282). SGLT-2 inhibitors cause fat reduction in MetS by preventing renal glucose reabsorption (283). SGLT-2 is predominantly expressed in the renal proximal tubules and facilitates the reabsorption of glucose back into the bloodstream (284). Inhibiting SGLT-2 with specific drugs prevents this reabsorption, leading to increased urinary glucose excretion (285). The resultant caloric loss contributes to a negative energy balance, prompting the body to utilize alternative energy sources, including stored fat (286). This process induces a state of lipolysis and beta-oxidation, promoting the breakdown of triglycerides into free fatty acids and ketone bodies for energy (287). Consequently, adipose tissue experiences a reduction in fat stores, contributing to overall fat loss in individuals with MetS (288). The additional benefits of improved glycemic control and reduced cardiovascular risk further position SGLT-2 inhibitors as a promising therapeutic avenue for addressing the complex metabolic disturbances associated with MetS (289).

#### 7.4.2 Proprotein Convertase Subtilisin/Kexin Type 9 inhibitors

Proprotein Convertase Subtilisin/Kexin Type 9 (PCSK9) inhibitors function by disrupting the degradation of low-density lipoprotein (LDL) receptors on the surface of liver cells (290). In MetS, PCSK9 levels are elevated, leading to increased degradation of these receptors and reduced clearance of LDL cholesterol from the bloodstream (291). PCSK9 inhibitors, often administered as monoclonal antibodies, block the action of PCSK9, preventing the degradation of LDL receptors. This results in an elevated number of functional receptors on liver cells, enhancing the clearance of LDL cholesterol (292).

#### 7.4.3 Glucagon-like peptide-1 receptor agonists

Glucagon-like peptide-1 (GLP-1) receptor agonists exert their molecular influence on fat reduction in MetS through multiple pathways (293). These drugs, designed to mimic the action of endogenous GLP-1, primarily target pancreatic islets, enhancing insulin secretion in a glucose-dependent manner (294). Elevated insulin levels suppress glucagon release, reducing hepatic glucose production. Beyond glycemic control, GLP-1 agonists play a pivotal role in appetite regulation and satiety (295). They act on the central nervous system, particularly the hypothalamus, to induce feelings of fullness, reducing caloric intake (296). Additionally, GLP-1 receptor activation delays gastric emptying, further contributing to postprandial glucose control and weight loss (297). Notably, GLP-1 agonists exhibit direct effects on adipose tissue by promoting lipolysis and inhibiting lipogenesis, leading to reduced fat accumulation (298). These multiple actions collectively position GLP-1 receptor agonists as promising therapeutic agents for fat reduction in MetS, addressing both glycemic control and weight management (299).

#### 7.4.4 Glucagon receptor antagonists

Glucagon receptor antagonists (GRAs) represent a class of pharmacological agents designed to modulate the activity of the glucagon receptor, which is a key player in glucose homeostasis and metabolic regulation (300). The glucagon receptor is primarily expressed in the liver, where it stimulates the production and release of glucose (301). GRAs act by binding to the glucagon receptor and inhibiting its downstream signaling pathways. One of the main effects of GRAs is a reduction in hepatic glucose output, which contributes to improved glycemic control (302). By blocking the action of glucagon, GRAs aim to lower blood glucose levels, especially in individuals with type 2 diabetes or MetS characterized by IR and dysregulated glucose metabolism (303). Additionally, GRAs may influence lipid metabolism, as glucagon has effects on lipolysis and fatty acid oxidation (304).

#### 7.4.5 Peroxisome proliferator-activated receptor (PPAR) modulators

Novel peroxisome proliferator-activated receptor (PPAR) modulators operate at the molecular level to address fat reduction in MetS by selectively targeting PPAR subtypes (305). These compounds typically interact with PPAR-alpha and PPAR-gamma receptors, influencing gene expression patterns associated with lipid metabolism and adipogenesis (306). Activation of PPAR-alpha enhances fatty acid oxidation, promoting the breakdown of stored triglycerides for energy (307). Simultaneously, modulation of PPAR-gamma impacts adipocyte differentiation and insulin sensitivity, contributing to improved metabolic parameters (308). By orchestrating these mechanisms, novel PPAR modulators aim to enhance lipid utilization, mitigate fat accumulation, and ameliorate IR. The nuanced actions on both lipid metabolism and insulin sensitivity make PPAR modulation a promising avenue for addressing the complex interplay of factors contributing to fat reduction and metabolic improvements in individuals with MetS (309).

#### 7.4.6 Fecal Microbiota Transplant

Fecal Microbiota Transplant (FMT), also known as Intestinal Microbiota Transplant is a novel non-pharmaceutical therapy for MetS treatment (310). FMT involves the transfer of fecal material from a healthy donor to a recipient with the aim of modulating the recipient’s gut microbiome (311). The procedure normally begins with the selection of a thoroughly screened and healthy donor whose fecal material undergoes processing, which usually involves dilution and filtration to obtain a liquid suspension. The resulting suspension is then administered to the recipient via colonoscopy, nasogastric or nasoenteric tube, or capsules. The rationale behind FMT lies in the central role of gut microbiota in metabolic processes, inflammation, and overall health (312). The mechanisms underlying the use of FMT in MetS treatment include the restoration of a diverse and balanced microbiome composition (313). Dysbiosis, the imbalance in the gut microbiota, is associated with MetS, and FMT seeks to address this imbalance. The transplanted microbiota contributes beneficial bacteria, promotes the production of short-chain fatty acids, modulates inflammation, and influences energy metabolism. While the precise molecular details are still under exploration, FMT holds promise as an innovative therapeutic avenue for MetS by harnessing the intricate interplay between gut microbiota and host physiology (314).

#### 7.4.7 Gene therapy

Gene therapy is also being explored for MetS treatment (315, 316). This involves the delivery of genetic material to target cells with the aim of correcting underlying genetic defects or modulating gene expression to suppress metabolic abnormalities (317). The procedure begins with the identification of specific genes associated with MetS components such as IR, dyslipidemia, or obesity (318). The therapeutic genetic material, commonly in the form of viral vectors or other delivery systems, is then introduced into the patient’s cells, aiming to either replace or supplement deficient genes, inhibit the expression of detrimental genes, or introduce new genetic material to alter cellular functionality (319). Gene therapy in MetS mainly works through the targeted alteration of key signaling pathways involved in glucose and lipid metabolism. For instance, manipulating the expression of genes involved in insulin signaling, adipocyte function, or lipid metabolism can enhance insulin sensitivity, regulate adipose tissue development, and modulate lipid profiles (320, 321). Additionally, gene therapy may target genes associated with appetite regulation, thermogenesis, or energy expenditure, addressing multiple areas of MetS (322).

#### 7.4.8 RNA interference

The therapeutic potential of RNA interference (RNAi) in metabolic diseases lies in its ability to selectively silence genes that are involved in pathogenic processes. By harnessing small RNA molecules to target specific messenger RNAs, RNAi can modulate the expression of genes associated with metabolic disorders, such as obesity, diabetes, and dyslipidemia (323). This precise and gene-specific approach holds promise for developing therapies that mitigate aberrant metabolic pathways, improve insulin sensitivity, and regulate lipid metabolism (324). RNAi-based interventions offer a novel and targeted strategy for addressing the molecular underpinnings of metabolic diseases, potentially paving the way for more effective and tailored treatments in the realm of metabolic health (325).

#### 7.4.9 Caloric Restriction Mimetics

Caloric Restriction Mimetics (CRMs), exemplified by compounds like resveratrol and rapamycin, represent a pharmacological strategy to replicate the health benefits associated with caloric restriction (326). Resveratrol activates sirtuins, particularly SIRT1, pivotal in cellular regulation. SIRT1 activation improves mitochondrial function, insulin sensitivity, and glucose homeostasis. Concurrently, resveratrol stimulates AMPK, a cellular energy sensor, enhancing energy balance and metabolic activity (327). On the other hand, rapamycin acts by inhibiting the mammalian target of rapamycin (mTOR), a regulator of cell growth and metabolism (328). This inhibition induces autophagy, facilitating the removal of damaged cellular components and enhancing metabolic efficiency. Furthermore, mTOR inhibition by rapamycin may improve insulin sensitivity and mitigate inflammation (329, 330).

#### 7.4.10 Non-invasive neuromodulation techniques

Non-invasive neuromodulation techniques for correcting MetS, such as transcranial magnetic stimulation (TMS) and transcutaneous vagus nerve stimulation (tVNS), employ external stimuli to alter neural activity (331). TMS, utilizing magnetic fields, induces electrical currents in specific brain regions, influencing the neural circuits governing appetite regulation and metabolic control. This leads to alterations in the activity of the hypothalamus, which is key in energy homeostasis (332). On the other hand, tVNS targets the vagus nerve, a key component of the autonomic nervous system. Stimulation of the vagus nerve through tVNS can also influence the neuroendocrine pathways, including the release of insulin and glucagon, thereby affecting glucose metabolism (333). Additionally, tVNS may modulate inflammatory responses associated with MetS (334).

#### 7.4.11 Bariatric surgery

Bariatric surgery, including procedures such as gastric bypass and sleeve gastrectomy, induces profound molecular and physiological changes contributing to the correction of MetS (335). These surgeries predominantly impact the gastrointestinal tract, leading to alterations in hormonal signaling and nutrient absorption (336). Gastric bypass involves creating a small stomach pouch and rerouting the small intestine, resulting in reduced food intake and nutrient absorption. Sleeve gastrectomy involves removing a portion of the stomach, limiting its capacity (337). Both procedures significantly affect the release of gut hormones, particularly incretins like glucagon-like peptide-1 (GLP-1) and peptide YY (338). The surge in GLP-1 enhances insulin secretion and sensitivity, promoting glucose control. Simultaneously, increased PYY levels contribute to appetite suppression and improved satiety (339).

Beyond hormonal changes, bariatric surgery influences adipose tissue biology. Post-surgery, adipose-derived hormones like leptin and adiponectin undergo alterations (340). Leptin, decreases, contributing to reduced appetite and increased metabolism (341). Simultaneously, adiponectin levels increase, enhancing insulin sensitivity (342). These changes collectively contribute to improved metabolic health by regulating energy balance and insulin responsiveness (343). These changes in adipose tissue biology, ultimately support weight loss and glycemic control (344).

Additionally, there are changes in gut microbiota composition due to surgery, these also influence metabolic processes (345). Bariatric surgery promotes a positive shift in gut microbiota composition by fostering beneficial bacteria growth (346). This enhances energy extraction, improves insulin sensitivity, and influences bile acid metabolism (347). These changes positively impact metabolic processes, aiding weight loss and glycemic control in individuals undergoing the surgery (348).

### 7.5 The controversies and inconsistencies in diagnosis and management of metabolic syndrome

Controversies and inconsistencies surrounding the diagnosis and treatment of MetS highlight the challenges in the current understanding and managing the condition (349). Discussed below are some of the controversies and inconsistencies in the diagnosis and management of MetS.

#### 7.5.1 Lack of standard metabolic syndrome diagnostic criteria

One contentious issue revolves around the diverse definitions and cut-off points used for MetS diagnosis. Various medical organizations and guidelines propose slightly different criteria, leading to inconsistency in identifying individuals with the syndrome (350). Several of these criteria were previously discussed under the definitions of MetS. For instance, the WHO definition requires glucose intolerance or insulin resistance plus two additional factors such as elevated blood pressure (≥140/90 mm Hg), dyslipidemia, central obesity, or microalbuminuria. The EGIR emphasizes elevated plasma insulin alongside two metabolic abnormalities, excluding those with diabetes, while the IDF requires central obesity plus any two of four other risk factors. The harmonized criteria define MetS as the presence of any three out of five specified components. Notably, there are also variations in the definition of hypertension, European guidelines use a threshold of ≥140/90 mm Hg, whereas U.S. guidelines consider ≥130/80 mm Hg, further contributing to diagnostic inconsistencies (46–49, 351, 352).

The absence of standardized diagnostic criteria raises concerns about accurate prevalence estimates and impedes efforts to compare research findings and develop universally applicable treatment strategies (353).

#### 7.5.2 Unanswered questions on high-density lipoprotein cholesterol-associated management strategies

The management of reduced HDL-cholesterol within MetS is marked by controversies within the medical community. Despite HDL-cholesterol’s traditional role as a “good” cholesterol in cholesterol transport, debates arise about whether raising HDL-cholesterol directly leads to improved cardiovascular outcomes (354, 355). Pharmaceutical interventions designed to increase HDL-cholesterol have shown limited success, prompting debates about the clinical significance of HDL-cholesterol as an independent therapeutic target (356). Additionally, debates emerge regarding the potential heterogeneity of HDL particles. Not all HDL particles exhibit the same functionality, challenging the simplistic view of HDL-cholesterol as a singular biomarker and raising questions about whether focusing solely on HDL-cholesterol levels adequately captures the intricacies of HDL’s function and its impact on cardiovascular health (357).

#### 7.5.3 Niacin use, risks vs. benefits

Controversies surround the use of niacin in MetS management, with conflicting perspectives on its efficacy and safety. Niacin has been traditionally employed to raise HDL-cholesterol levels, an essential consideration in MetS (358). However, recent studies have cast doubt on its overall cardiovascular benefits, particularly concerning its ability to reduce cardiovascular events (359, 360). Additionally, concerns about side effects, including flushing, hepatotoxicity, and glucose intolerance, have led to a reevaluation of niacin’s role in the comprehensive treatment of MetS (361, 362).

#### 7.5.4 Risks associated with use of thiazolidinediones

Thiazolidinediones have been subject to controversy primarily due to concerns about their safety profile. Rosiglitazone, a widely prescribed TZD, faced scrutiny due to studies suggesting an increased risk of cardiovascular events, leading to regulatory restrictions on its use (363). Additionally, both rosiglitazone and pioglitazone, another TZD, have been associated with concerns about fluid retention, weight gain, and an elevated risk of fractures (364, 365). While TZDs effectively improve insulin sensitivity and glycemic control, potential cardiovascular and safety issues have fueled debates about their overall risk-benefit ratio (366).

#### 7.5.5 Bariatric surgery; questions on long-term effectiveness and risks

Bariatric surgery as a therapeutic option for MetS is another area of controversy (367). While some studies suggest significant improvements post-surgery, questions persist about such interventions’ long-term effectiveness and safety (368). Debates within the medical community revolve around the potential complications and the necessity of surgery compared to less invasive treatments (369).

#### 7.5.6 Fecal Microbiota Transplant; ethical issues and potential risks

Fecal Microbiota Transplant is an innovative therapeutic approach for MetS, however, uncertainties surrounding FMT’s long-term safety, efficacy, and standardization have triggered significant debate (370, 371). Ethical considerations related to the manipulation of the microbiome, potential unintended consequences, and the necessity for rigorous clinical trials have added complexity to the discussion (310, 372).

#### 7.5.7 Dilemma on diagnosis and management of metabolic syndrome in children

There are also concerns surrounding the diagnosis and treatment of MetS in children. Key points of contention include the definition and diagnosis of MetS in children, posing challenges due to developmental variations and differences from adult criteria. Applying adult criteria to pediatric populations raises concerns about overdiagnosis and overtreatment (373). The primary approach for managing MetS in children involves lifestyle modifications, but controversies arise regarding the potential need for pharmacological interventions (374). Ethical considerations play a significant role, involving informed consent, autonomy, and the impact of interventions on a child’s well-being. Controversies emerge regarding the role of parents, healthcare providers, and the child in decision-making, especially concerning lifestyle changes and, in some cases, medication use (375).

#### 7.5.8 Future perspectives

Advancing our understanding of the molecular mechanisms that govern MetS is essential for the development of highly effective interventions. Future research need to focus on understanding the interrelationships between genetic susceptibility, epigenetic regulation, and environmental exposures that drive metabolic dysfunction. The identification of reliable biochemical markers, such as inflammatory cytokines, adipokines, metabolites, and circulating microRNAs, holds promise for improved early diagnosis and patient risk stratification. Additionally, the integration of genomic data, including the use of polygenic risk scores (PRS), may enable the identification of individuals genetically predisposed to MetS, even before clinical manifestations emerge (376, 377). Such approaches could support personalized prophylactic strategies and inform tailored therapeutic regimens. Importantly, nutrigenomic research may clarify how specific dietary patterns and nutrients modulate gene expression and metabolic pathways, offering opportunities for individualized dietary interventions based on genetic and metabolic profiles. Progress in molecular phenotyping and systems biology is expected to accelerate the development of targeted therapies that modulate key molecular pathways implicated in insulin resistance, lipid metabolism, and low-grade inflammation. Ultimately, translating these valuable insights into clinical practice could reshape MetS management through early detection, risk prediction, and personalized nutrition and pharmacological intervention.

## 8 Conclusion

Metabolic syndrome prevalence is on the rise worldwide, and it is an important public health challenge. The underlying mechanisms of MetS are complex and involve insulin resistance, obesity, dyslipidemia, hypertension, inflammation, oxidative stress, and endothelial dysfunction. Lifestyle changes, such as dietary changes, increased physical activity, smoking cessation, and alcohol reduction, are the pillars of managing MetS. Evidence-based eating patterns, such as the Mediterranean and DASH diets, have shown strong benefits in reducing insulin resistance, inflammation, and cardiometabolic risk. Pharmacological interventions may complement lifestyle changes in managing individual components. Medications may also be included to control individual risk factors. Further research is needed to develop targeted therapeutic interventions for MetS. Promoting awareness of lifestyle changes and early screening can help reduce the prevalence and impact of MetS at the individual, family, community, and health-system levels.
